# Correlated Allele Frequency Changes Reveal Clonal Structure and Selection in Temporal Genetic Data

**DOI:** 10.1093/molbev/msae060

**Published:** 2024-03-20

**Authors:** Yunxiao Li, John P Barton

**Affiliations:** Department of Physics and Astronomy, University of California, Riverside, CA 92521, USA; Department of Physics and Astronomy, University of California, Riverside, CA 92521, USA; Department of Computational and Systems Biology, University of Pittsburgh School of Medicine, Pittsburgh, PA 15260, USA

**Keywords:** Bayesian inference, clonal interference, linkage disequilibrium, selection, temporal genetic data

## Abstract

In evolving populations where the rate of beneficial mutations is large, subpopulations of individuals with competing beneficial mutations can be maintained over long times. Evolution with this kind of clonal structure is commonly observed in a wide range of microbial and viral populations. However, it can be difficult to completely resolve clonal dynamics in data. This is due to limited read lengths in high-throughput sequencing methods, which are often insufficient to directly measure linkage disequilibrium or determine clonal structure. Here, we develop a method to infer clonal structure using correlated allele frequency changes in time-series sequence data. Simulations show that our method recovers true, underlying clonal structures when they are known and accurately estimate linkage disequilibrium. This information can then be combined with other inference methods to improve estimates of the fitness effects of individual mutations. Applications to data suggest novel clonal structures in an *E. coli* long-term evolution experiment, and yield improved predictions of the effects of mutations on bacterial fitness and antibiotic resistance. Moreover, our method is computationally efficient, requiring orders of magnitude less run time for large data sets than existing methods. Overall, our method provides a powerful tool to infer clonal structures from data sets where only allele frequencies are available, which can also improve downstream analyses.

## Introduction

Clonal interference refers to competition between subpopulations with different beneficial mutations. This phenomenon can occur in populations with sexual reproduction, but it is especially common in asexually reproducing populations without recombination. In such populations, individuals can be grouped into competing clades or lineages, which are defined by shared sets of mutations. Clonal interference is common in a wide range of microbial and viral populations because of their larger population sizes and higher mutation rates. For example, long-term coexistence of competing clades is found in experimental populations of *Escherichia coli* (Good et al. 2017). Strong clonal interference has been observed in the evolution of influenza A (H3N2) in time-series data collected over 39 years (Strelkowa and Lässig 2012). Other examples include yeast (*Saccharomyces cerevisiae*; Lang 2013), the malaria parasite *Plasmodium falciparum* (Jett et al. 2020), and HIV-1 viruses (Pandit and de Boer 2014).

However, it can be difficult to fully recover clonal dynamics from data. To achieve high throughput and low cost, next generation sequencing techniques generally involve randomly breaking a large number of genomes into smaller fragments and sequencing them in parallel (Metzker 2010). Estimates of individual allele frequencies can then be obtained by mapping the generated short reads to a reference genome. While this approach allows for excellent estimates of allele frequencies, full haplotypes and a complete picture of linkage disequilibrium (LD) are generally lost.

Challenges in resolving clonal structure also make it more difficult to quantify the selective forces driving population evolution. Due to clonal interference, the fate of a mutation depends not only on its individual fitness effect but also on the fitness of the genetic background on which it appears. For example, even highly beneficial mutations can be outcompeted if they occur on a deleterious genetic background. Theoretical and experimental studies have shown that clonal interference extends the time required for mutations to fix and increases genetic diversity, among other effects (Park and Krug 2007; Fogle et al. 2008; Wiser et al. 2013; Maddamsetti et al. 2015; Harris et al. 2021; Guo and Amir 2022). Past work has also indicated that accounting for LD (due, for example, to clonal interference) is important to accurately estimate the fitness effects of individual mutations from data (Sohail et al. 2021). There are multiple methods that can use LD or haplotype frequencies to improve estimates of selection when such data are available (Illingworth and Mustonen 2011; Illingworth et al. 2014; Terhorst et al. 2015; Sohail et al. 2021).

These challenges have motivated research into inferring haplotypes or LD from short-read sequencing data (Beerenwinkel et al. 2012). Some approaches use overlaps among short reads to assemble them into haplotype sequences that span the entire genomic region of interest (Zagordi et al. 2011). Others take time-series allele frequencies as input and infer LD or haplotype information from evolutionary dynamics (Franssen et al. 2017; Barghi 2019; Deitrick 2020; Pelizzola et al. 2021; Shen et al. 2021; Li and Barton 2023). For example, *haploSep* uses time-series allele frequency data to infer haplotype frequencies for evolving populations with stable haplotype structures (Pelizzola et al. 2021). *Evoracle* is a machine learning method that reconstructs full-length haplotype frequency trajectories and fitness from time-series allele frequency data generated by directed evolution campaigns (Shen et al. 2021). *Lolipop* (Deitrick 2020) clusters allele frequency trajectories based on measures of similarity and reconstructs haplotypes and their frequency trajectories. Previously, we provided a generic method to estimate LD from time-series allele frequency data with sufficiently dense temporal sampling (Li and Barton 2023). The *haploReconstruct* method was developed to automatically identify selected haplotype blocks from temporal allele frequencies using correlation coefficients between normalized trajectories as a measure of their LD (Franssen et al. 2017; Barghi 2019).

However, populations with significant genetic diversity could present a challenge to computational methods based on haplotype reconstruction. The number of possible haplotypes grows exponentially with the number of mutations, making it challenging to explore the space of haplotypes and estimate haplotype frequencies. In such cases, methods that rely on pairwise statistics (e.g. clustering of allele frequency trajectories) may remain more computationally tractable for highly diverse populations.

Here, we present a method that uses pairwise allele frequency statistics to infer clonal structure from time-series sequence data. We assume that the population consists of groups of alleles that evolve collectively as clades in the absence of recombination (later, we will consider relaxing these assumptions). We define the inference of clonal structure as inferring the number of clades in the population, estimating their time-varying frequencies, and recovering clonal identities (i.e. which clade(s) does an allele belong to) for all alleles. We view clades as families of closely related haplotypes with shared alleles. Reconstructing clades and their dynamics thus gives a more coarse-grained view of the population than approaches that aim to precisely recover haplotypes. Our approach works by examining the matrix of correlations between allele frequency changes over time, where we assume that changes in allele frequency will tend to be positively correlated for mutations on the same genetic background and negatively correlated for mutations on competing genetic backgrounds.

Based on the correlation matrix of allele frequency changes, we classify alleles into a number of clades and estimate the fraction of the population represented by each clade at each time. We then use the recovered clonal structure to estimate LD for each pair of alleles: alleles that belong to the same clade are likely to be positively correlated and alleles belonging to competing clades are likely to be negatively correlated. LD estimates can then be used by inference methods such as marginal path likelihood (MPL; Sohail et al. 2021) to improve estimates of the fitness effects of individual alleles. Our assumption that the population consists of competing clades allows us to approach the problem of recovering evolutionary dynamics at an intermediate level of detail, without attempting to explicitly reconstruct all haplotypes.

Simulations and tests on real data show that our method recovers the clonal structure of evolving populations when they are known. This allows for accurate estimates of LD and improves inference of the fitness effects of individual mutations. Applying our method to data from the *E. coli* long-term evolution experiment (LTEE) reveals potential clonal structure beyond previous descriptions. Tests on data from bacterial parallel evolution experiments also allow us to make better inferences about the effects of mutations on bacterial fitness and antibiotic resistance than with alternative methods. The method we describe is computationally efficient, requiring orders of magnitudes less run time than alternative methods that aim to reconstruct full-length haplotypes. Overall, our method provides a powerful tool to infer clonal structures from short-read sequencing data with only allele frequencies available. In turn, this enables the use of linkage-aware methods for inferring selection, which are able to better recover underlying fitness values than methods that ignore LD.

## Results

### Method Overview

**Fig. 1.**
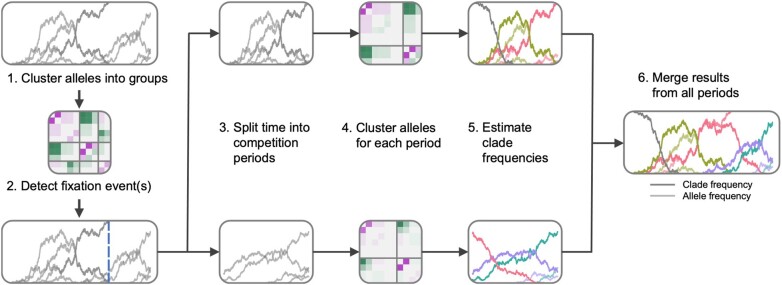
An overview of the method. We first compute the *D* matrix and cluster alleles into groups according to *D* matrix. The clustering results are reflected by the heatmap of the rearranged and segmented matrix *D*, where each block corresponds to a group. Aside from the group that consists of shared alleles which may or may not exist, each group consists of alleles that collectively compete with other groups. We then detect fixation events of alleles that are not shared. Once detected, we split time into competition periods at the fixation time(s). (*n* fixation events will result in n+1 competition periods.) We cluster alleles for each period. We then take the groups as initial clades and iteratively refine clonal identities and clade frequencies. Finally, we merge results from all periods into a complete reconstruction of the clonal interference throughout the evolution. The allele frequency trajectory data plotted in the figure are from a simulation, where a population of 1,000 sequences were simulated to evolve for 1,000 generations.

Here we consider an evolving population consisting of a number of clades, which are defined by shared mutations. We will typically think of these “mutations” as single nucleotide polymorphisms, but more complicated genetic alterations such as duplications, deletions, inversions, or translocations could be treated as an “allele” in the same way. We assume that each allele has a fixed identity: it is either exclusively possessed by a single clade or shared among two or more clades, and this property remains unchanged during the period of clonal interference. We first cluster all alleles into clades based on how their frequencies co-vary, quantified by the matrix of products of changes in allele frequencies over time (Fig. 1). Clades and their frequencies are then recursively refined following a metric for clade reconstruction quality that accounts for both co-varying allele frequencies and sampling probabilities. Finally, we compute measures of LD over time based on the recovered clonal structure and use these data to infer selection coefficients with MPL.

#### Quantifying Correlated Allele Frequency Changes

Intuitively, alleles that belong to the same clade are likely to experience changes in frequency that are positively correlated, while alleles on different backgrounds are more likely to change in ways that are negatively correlated (for example, due to the growth of one clade at the expense of another). To quantify this, for time-series genetic data sampled at times $t_{1},t_{2},\ldots ,t_{K}$, we consider the matrix of products of allele frequency changes ΔxΔx, which has entries


$$\Delta x\Delta x_{ij}(t_{k})=(x_{i}(t_{k+1})-x_{i}(t_{k}))(x_{j}(t_{k+1})-x_{j}(t_{k})).$$

Here, $x_{i}(t_{k})$ denotes the frequency of allele *i* at time $t_{k}$. For other genetic alterations (deletions, etc.), $x_{i}(t_{k})$ represents the fraction of haplotypes in the population at time $t_{k}$ that have feature *i*. If two alleles *i* and *j* both increase or decrease in frequency at generation $t_{k}$, then $\Delta x\Delta x_{ij}(t_{k})$ will be positive. However, if one allele increases in frequency while the other decreases, then $\Delta x\Delta x_{ij}(t_{k})$ will be negative. In this way, the sign and magnitude of the entries of ΔxΔx quantify correlated frequency changes for different alleles. Because of the central role of the product of allele frequency changes in our analysis, we refer to our method as *dxdx* below.

To reduce the influence of finite sampling noise on observed changes in allele frequencies, we weight ΔxΔx values by allele frequency variances and compute their sums over time (supplementary material Methods, Supplementary Material online). We refer to this rescaled matrix as *D*. To prevent confusion, we emphasize that this matrix is not the same as the matrix of LD values (Hedrick 1987), which is sometimes also written as *D*.

#### Forming Initial Clades

To form clades, we aim to construct groups of alleles such that alleles exhibit cooperating behavior (defined as having positive entries in *D*) within each clade, and exhibit competing behavior (negative entries in *D*) across clades. For each allele *i*, we quantify its cooperating behavior with a group of alleles, *g*, by a cooperation score, $\rho_{\mathrm{coop}}$, computed as the mean *D* entries of itself and each allele in that group,


$$\rho_{\mathrm{coop}}(i,g)=\frac{1}{N_{g}}\sum_{\;j\in \text{group}\;g}D_{ij},$$

where $N_{g}$ is the number of alleles in group *g*. When $\rho_{\mathrm{coop}}(i,g)$ is positive, allele *i* is considered to cooperate with group *g*. When it is negative, allele *i* is considered to compete with group *g*.

We begin sorting alleles into clades by identifying the pair of alleles that appear to compete the most (i.e. the ones with the most negative entry in *D*) and assigning those as members of the first two clades. We then proceed through all the remaining alleles, classifying them as a member of an existing clade if they cooperate with that clade and compete with others, or as a shared allele if they cooperate with multiple clades (supplementary material Methods, Supplementary Material online). Alleles that compete with all existing clades can form a new clade.

#### Iterative Refinement of Clade Membership and Frequencies

During the course of evolution, a population can exhibit different patterns of clonal interference at different times. For example, consider a population with multiple clades, where one of the clades ultimately outcompetes the others and fixes. At a later time, new beneficial mutations can arise on different backgrounds and compete with one another, initiating another period of clonal interference.

To account for this possibility, we detect times when alleles fix and then split the time interval into different “competition periods” that feature different clonal structures (Fig. 1). We then repeat the steps described above for each competition period, iteratively assigning alleles to clades and estimating clade frequencies, before merging the results for all competition periods together (supplementary material fig. S1, Supplementary Material online). Full details of this procedure are described in supplementary material Methods, Supplementary Material online.

#### Estimating LD

Estimates of clade frequencies and the alleles that belong to each clade can be used to estimate LD. Specifically, we are interested in the allele frequency covariance matrix, which is a measure of LD (Hedrick 1987) and is defined as


(1) $$C_{ij}(x(t))\;:=\left\{ \begin{array}{cc} x_{i}(t)(1-x_{i}(t)), & i=j,\\ x_{ij}(t)-x_{i}(t)x_{j}(t), & i\neq j. \end{array}\right.$$

Here, $x_{ij}(t)$ is the frequency of haplotypes in the population with mutant alleles at sites *i* and *j* at time *t*.

To estimate LD, we first assume that a pair of alleles *i* and *j* are in linkage equilibrium ($C_{ij}(t)=0$) if one or both of the alleles is classified as a shared mutation. For alleles that belong to the same clade, we assume $x_{ij}(t)=\min(x_{i}(t),x_{j}(t))$, and for alleles that are in competing clades, we set $x_{ij}(t)=\text{max}(0,x_{i}(t)+x_{j}(t)-1)$.

#### Inferring Selection with MPL

Together with the allele frequencies themselves, we can use estimates of LD to infer the fitness effects of individual mutations using methods such as MPL (Sohail et al. 2021, 2022). MPL is a framework for statistical inference of selection from evolutionary histories. While originally developed in the context of population genetics, it has also been recently applied to other problems (Shimagaki and Barton 2023), including disease transmission in epidemiological models (Lee 2022). The main idea of this approach is to estimate a set of selection coefficients for individual alleles that best explain an observed evolutionary history, in the sense that these selection coefficients maximize the posterior probability of the data. In MPL, this is accomplished using a diffusion approximation (Ewens 2012) for the likelihood, ultimately yielding an analytical expression for the maximum *a posteriori* selection coefficients


(2) $$\begin{array}{rl} \hat{s} & ={\left[\sum_{k=0}^{K-1}\Delta t_{k}C(x(t_{k}))+\gamma I\right]}^{-1}\\ & \;\times \left[x(t_{K})-x(t_{0})+\mu \sum_{k=0}^{K-1}\Delta t_{k}(2x(t_{k})-1)\right]. \end{array}$$

Here, $\Delta t_{k}=t_{k+1}-t_{k}$, *μ* is the mutation rate, $x(t_{k})$ is the vector of mutant allele frequencies at time $t_{k}$, and γI is a multiple of the identity matrix serving as a regularization term. In a Bayesian sense, the regularization term γI can be interpreted as a Gaussian prior distribution over the selection coefficients with zero mean and 1/γN variance. We set γ=1 by default, which slightly constrains magnitudes of inferred selection coefficients and helps to ensure that the matrix term is invertible. A more detailed introduction to MPL can be found in the supplementary material Methods, Supplementary Material online.

MPL assumes simple, directional selection, which may not apply in all cases. For example, some alleles may exhibit frequency-dependent selection. Different clades may also occupy distinct ecological niches which prevent them from directly competing with one another. Such cases do not pose an additional challenge for clade reconstruction, but they should be considered when interpreting the selection coefficients inferred by MPL.

### Validation in Simulations

To benchmark the performance of our method, we generated artificial time-series sequence data by simulating the evolution of a population of N=1,000 haploid individuals under an infinite sites model. In this model, each mutation happens on a unique site, with a total mutation rate $\mu =2\times 10^{-4}$ per sequence per generation. We assume that the probability of back mutations (i.e. a mutant allele reverting to wild-type) is zero, and that there is no recombination. These assumptions generate a population with a strong clonal structure. Selection coefficients for each new mutation are drawn from a Gaussian distribution centered at 0.03 with a standard deviation of 0.01. In the analysis described below, we filtered the resulting sequence data from simulations to remove mutations that never exceeded a frequency of 5%.

#### Recovering Clonal Structure

**Fig. 2.**
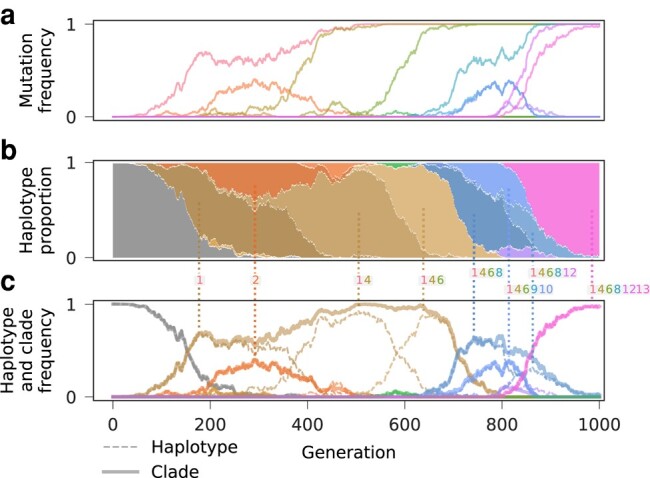
Clonal structure is accurately recovered from simulated allele frequency trajectories. a) Allele frequency trajectories for 13 mutations that occurred in an example simulation and exceeded a frequency threshold of 5%. Six mutations eventually fixed in the population, while the rest were lost. These data serve as input for our method. b) The succession of true, underlying haplotype frequency trajectories. Haplotypes inferred to be in the same clade are colored with the same hue and saturation, but different brightness. c) True haplotype frequency trajectories versus reconstructed clade frequency trajectories. Reconstructed clades can consist of a single haplotype, e.g. the final dominant clade, or several haplotypes that emerge consecutively on top of their predecessors.

Figure 2 shows an example of our clade reconstruction from simulated data. Based on allele frequency trajectories, our method is able to identify groups of mutations that compete collectively as clades. Reconstructed clades clearly match in identity and frequency with individual haplotypes or groups of haplotypes that emerge successively from a common ancestor (Fig. 2c).

#### Recovering LD, Selection Coefficients, and Fitness

**Fig. 3.**
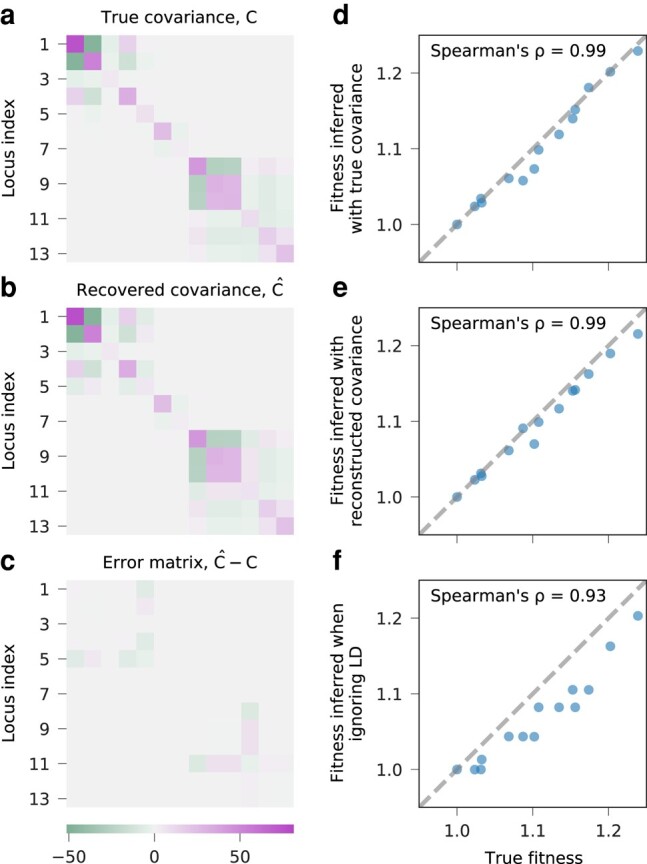
Allele frequency covariance and fitness are accurately inferred from recovered clonal structure. a) True and b) recovered integrated allele frequency covariance matrices, and c) their difference are plotted for the simulation example in Fig. 2. The fitness of all haplotypes present in the evolution, inferred with d) the true covariance, e) covariance recovered with *dxdx*, and f) only variances (ignoring LD) are compared against true fitness values. Both allele frequency covariance and fitness values are accurately recovered. In this example, the fitness values inferred when ignoring LD are also strongly correlated with true values, but not as accurate as those inferred with our method.

As shown in Fig. 3, the reconstructed clade competition from our method is typically able to provide an accurate estimate of the allele frequency covariance matrix and improve inference of fitness values. We benchmarked the performance of our method on 40 simulations and compared our results with estimates of selection obtained using equation (2) with the true covariance matrix. The true, underlying covariance matrix is not available in pool-sequenced data, and so this approach can be viewed as an ideal limit for optimal performance. Our method, which we refer to as *dxdx*, uses the covariance matrix computed with inferred clonal structures.

We further compared our method with three alternatives that use time-series allele frequencies as input. They either directly estimate allele frequency covariance information (Li and Barton 2023) or reconstruct haplotypes and their time-series frequencies (Deitrick 2020; Pelizzola et al. 2021; Shen et al. 2021), which also provide covariance information. *Lolipop* evaluates the pairwise similarity of all allele frequency trajectories, clusters similar alleles into genotypes, and then nests successive genotypes (Deitrick 2020). *Evoracle* first proposes a number of possible haplotypes present in the evolution based on the observed allele frequency trajectories, then infers haplotype frequency trajectories by optimizing a loss function with gradient descent (Shen et al. 2021). The loss function includes a data-fitting term that measures how allele frequencies are recovered, a fidelity term that measures how the genotype frequency trajectories follow fitness-based dynamics, and a regularizer (Shen et al. 2021). The *LB* method directly estimates covariance at each time with the matrix of products of allele frequencies changes (Li and Barton 2023).

**Fig. 4.**
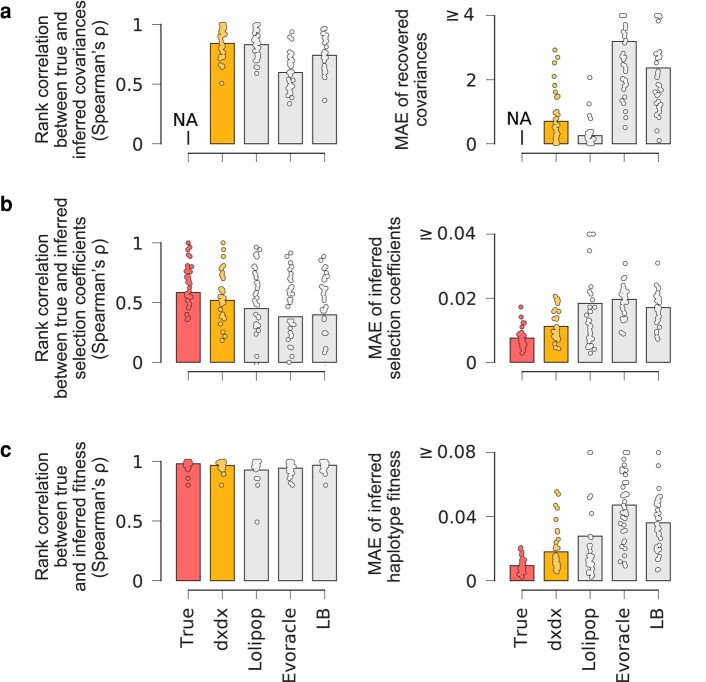
Recovered clonal structure improves inference of selection in simulated data. Performance of five methods for inference of a) integrated allele frequency covariance, b) selection coefficients, and c) haplotype fitness. The left column shows rank correlations with true values, and the right column shows the mean absolute error (MAE) of inferred values versus true values. The *True* method uses the true allele frequency covariance matrix, which is not available in short-read data, and represents the ideal performance.

Figure 4 shows that our method provides the best results among all methods that do not use true covariance information for inferring selection coefficients and fitness values, in terms of both rank correlation (Spearman’s *ρ*) and mean absolute error (MAE). Correlations between true and inferred allele frequency covariances are roughly equal between *dxdx* and *Lolipop*, and *Lolipop* tends to have lower MAEs for this quantity. However, this does not always yield better estimates of selection.

Due to the regular succession of more beneficial mutants, inferring the correct order of haplotypes’ fitness values is not difficult in our simulations. However, inferring the correct order among selection coefficients is much more challenging. Multiple beneficial mutations can emerge and fix within a clade quickly, after which they are linked indefinitely. Thus, the time period containing information that can separate each mutation’s effect is limited. This is why all methods yield high correlations for inferred fitness values, but much lower correlations for inferred selection coefficients. While accurate inference of fitness values for haplotypes is sufficient to explain the observed evolution itself, inferring selection coefficients is important because it allows for predicting the fitness of haplotypes unseen in existing data, and for explaining the underlying drivers of fitness.

#### Effects of Different Temporal Sampling Intervals

Our method uses changes in allele frequencies at consecutive sampling time points to quantify the relationship between each pair of alleles. To test its dependence on sampling intervals, we subsampled the simulated data using different time gaps Δg between samples. We then compared the performance of our method and alternatives for Δg=1,10,20,50, and 100 generations in supplementary material fig. S2, Supplementary Material online. Patterns of recovered covariances (evaluated by rank correlation coefficients and MAE) are similar to those shown in Fig. 4, except that *Evoracle* improves relative to other approaches for larger time gaps. In terms of inferring the correct value of selection coefficients and haplotype fitness, our method displays better performance for Δg<20, while *Evoracle* gains a slight advantage over other approaches for Δg>20. Interestingly, we found that the performance of *Evoracle* tends to improve when data are sampled less frequently, hitting a maximum in the range of Δg∼ 10–50 generations before declining with larger time gaps.

#### Performance Under Different Evolutionary Scenarios

In developing our approach, we made several simplifying assumptions, focusing on haploid genomes and assuming no recombination. Our initial tests in simulations also assumed that all individuals are identical in the starting population. In this section, we consider alternative scenarios and explore the effects of changing the population size.

First, we assessed the performance of our approach and alternatives at both smaller (N=100) and larger (N=10,000) population sizes. A smaller population size places greater emphasis on genetic drift, while a larger population size de-emphasizes drift and increases the supply of mutations. For the smaller population size, we find overall patterns for the recovery of LD that are qualitatively similar to those for the N=1,000 case, but the recovery of individual selection coefficients is more difficult (supplementary material fig. S3, Supplementary Material online). For the larger population size, patterns of recovered LD are again similar (supplementary material fig. S4, Supplementary Material online). However, methods that rely on haplotype reconstruction have more difficulties in revealing underlying selection for the simulations with larger population sizes. This appears to be related to the challenge of reconstructing haplotypes in a more genetically diverse population. Errors in reconstructing haplotypes then lead to spurious correlations between alleles, which can skew estimates of selection.

Next, we considered the effects of occasional recombination on our results by introducing a recombination probability $r=10^{-6}$ per replication event. This results in an average of 175 recombination events per simulation. Supplementary material fig. S5, Supplementary Material online, shows that *dxdx* performs quite well in this case, even though we assumed no recombination in developing our approach. Overall, the results in this case are similar to those in Fig. 4, but with occasional larger errors for all methods.

We also performed simulations of diploid populations with random mating. In this case, “clades” represent haplotypes with shared genetic variation. For simplicity, we set dominance to 0.5 for determining selection. While this choice does not affect the reconstruction of clades, different choices for dominance would affect our interpretations of inferred selection. In this scenario, the results across all methods were similar to those observed in the previous case with rare recombination (supplementary material fig. S6, Supplementary Material online).

Finally, we performed simulations including more extensive standing variation, rather than starting with a clonal population. Here we start simulations with a random combination of 3–11 haplotypes containing 30 non-shared mutations in total. The initial haplotype frequencies are then selected uniformly at random. Despite somewhat larger errors on inferred LD, *dxdx* performs well in this scenario, with low errors on inferred selection coefficients and haplotype fitness values (supplementary material fig. S7, Supplementary Material online). Here *Lolipop* also performs particularly well compared to other simulations.

### Applications to Temporal Genetic Data

Here we apply our approach to several temporal genetic data sets. When applied to data from the *E. coli* LTEE, we find some patterns of clade competition that are consistent with past work (Good et al. 2017) and, because we consider the possibility of multiple clades, some that are novel. We also study data from a pair of parallel evolution experiments (Scribner et al. 2020; Harris et al. 2021), where our approach yields inferred fitness values that match well with those measured experimentally.

#### Reconstructing Long-Term Clade Competition in LTEE Data

The *E. coli* LTEE has propagated 12 populations of *E. coli* in the same environment for more than 60,000 generations (Lenski et al. 1991). Prominent patterns of clonal interference have been observed in 9 out of these 12 populations. Previous work developed a hidden Markov model to assign mutations to basal, major, or minor clades, and to infer their frequencies over time (Good et al. 2017).

This work showed that the coexistence of multiple clades is sustained for over 10,000 generations in some populations, during which mutations continue to fix in each clade. The remarkable difference between timescales of within-clade and population-wide fixation events is difficult to explain by clonal interference, and instead is likely driven by ecological interactions. For example, it has been demonstrated that negative frequency-dependent selection exists in the population m2 and can explain the sustained coexistence of two clades (Rozen and Lenski 2000; Plucain 2014). Regardless of the mechanism of coexistence, the LTEE provides a valuable data set to test the ability of our method to recover clonal structure.

**Fig. 5.**
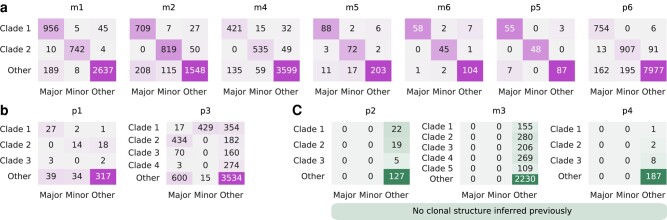
Clustering results are consistent with previous results for most populations. a) Cases where clonal structure inferred by our method is clearly consistent with previous analysis. b) Divergent results are obtained for populations p1 and p3. Here, clustering for p3 was obtained by splitting the trajectory into two periods and merging the results (supplementary material fig. S8, Supplementary Material online). c) For three populations, we infer some clonal structure where none was previously detected.

**Fig. 6.**
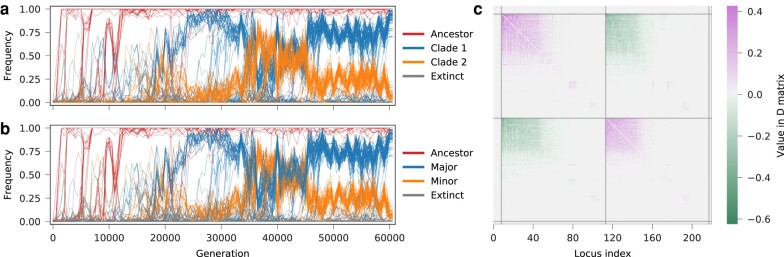
Inferred clonal structure for population m6 of LTEE data is consistent with previous analysis. The clustering results from a) our method and b) previous results (Good et al. 2017) on the population m6 are almost identical. Both results indicate that m6 features long-term coexistence and competition between two clades. c) *D* matrix segmented into groups during the initial clustering process. The competition between two clades is reflected clearly in the *D* matrix as two major blocks of entries. Alleles that belong to the same clade have frequency changes that are positively correlated with one another (positive *D* values), while those in competing clades have anti-correlated frequency changes (negative *D* values).

Figure 5 shows the clonal structure that we recover across LTEE populations. Initial analyses treated the entire trajectory as one competition period due to the difficulty of automatically determining period boundaries with the large number of alleles in this data set. Our results match very closely with previous findings (Good et al. 2017) in 7 of 12 cases. As one example, Fig. 6 shows clades inferred by our approach for population m6, which are almost identical to those obtained in Good et al. (2017). This lends additional support to the previously inferred clonal structure in these cases, as our analysis does not specify *a priori* the number of clades to cluster alleles into.

**Fig. 7.**
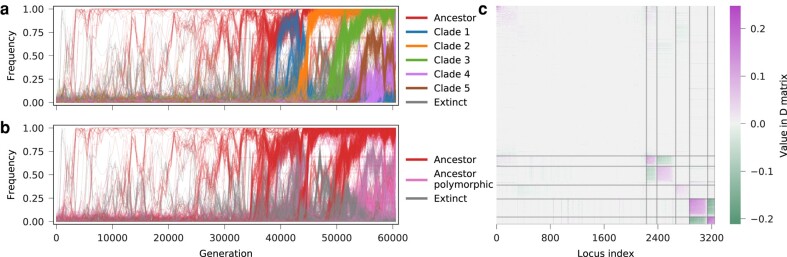
Evidence of clonal structure for population m3. a) Our method suggests patterns of clade competition around generation 42,000 (between clade 1 and clade 2) and during the last 5,000 generations (between clade 4 and clade 5), b) in contrast with previous results (Good et al. 2017). c), The *D* matrix segmented into groups during the clustering process. We can see prominent competition signals (negative entries) between clade 1 and clade 2, and between clade 4 and clade 5 in the *D* matrix. The population m3 is one of the mutator populations which have notably higher mutation rates and more mutations than other populations.

1wIn two cases we infer clonal structure that differs more substantially from those in previous work (Good et al. 2017). One such example is population p3, which displays complex clonal dynamics that are difficult to fully resolve (supplementary material fig. S8, Supplementary Material online). In the final three cases, we find evidence for some clonal structure where none was previously inferred. For example, in population m3, we observe competition between sub-clades of one dominant lineage (Fig. 7). The clonal structure that we infer for population p4 is subtle, however, so this case also displays good agreement with past analysis (Good et al. 2017).

#### Inferring Clonal Structure and Fitness from Parallel Evolution

**Fig. 8.**
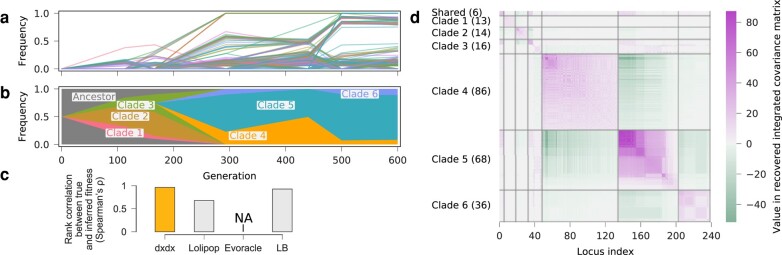
Clonal structure and fitness inference for B1 replicate of *P. aeruginosa* evolution data. a) Mutant allele frequency trajectories and b) clade frequency trajectories inferred by our method. c) We find a strong correlation between our fitness estimates and experimental measurements, exceeding those from *Lolipop* and similar to results following the *LB* method. Applying *Evoracle* to this data set yielded NaN results. d) The recovered integrated covariance matrix segmented into blocks according to clustering results of our method. The number of alleles in each clade is shown in brackets. Alleles in the same clades tend to show cooperating behaviors, as indicated by positive entries. Alleles tend to show competing behaviors across clades, as indicated by negative entries.

We applied our method to recover clonal structure and infer fitness from time-series allele frequency data from two parallel evolution experiments (Scribner et al. 2020; Harris et al. 2021). In the first experiment, six populations of *P. aeruginosa* strain PA14 were propagated for 90 days (600 generations; Harris et al. 2021). Time-series allele frequencies, obtained by longitudinal whole-population genome sequencing, revealed high genetic diversity that is sustained through the end of the 90-day evolution (Fig. 8a). This genetic diversity was due to the prevalence of mutator alleles, which increase genome-wide mutation rates, and clonal interference among multiple lineages, which slows down the fixation of fitter haplotypes.

We applied our method to this data set to recover clonal structure, infer selection coefficients, and compute fitness values for all the evolved populations at the end of the 90-day evolution. Figure 8 shows the recovered clonal structure and estimated covariance matrix for one of the six populations (B1), and performance on inferring population fitness compared with three other methods. Here, fitness was measured experimentally through competition assays (Harris et al. 2021). Applying *Evoracle* to this data set resulted in an error (i.e. NaN values), which meant that the correlation between experimentally measured and inferred fitness values could not be computed for this method. Among the other approaches, *dxdx* provided the highest correlation with experimental fitness values.

In a second parallel evolution experiment, two bacterial species from different families, *A. baumannii* and *P. aeruginosa*, were propagated for 12 days (80 generations) in media with increasing concentrations of tobramycin (TOB; Scribner et al. 2020). The experiment begins with TOB-sensitive ancestor clones. After 12 days of evolution under TOB selection, the populations exhibit higher TOB resistance levels relative to the ancestral clones, quantified by larger values of minimum inhibitory concentration (MIC) of TOB. Lineages with different driver mutations are found to compete with each other during evolution. MIC values for different genotypes were then measured from isolated clones using whole-genome sequencing (Scribner et al. 2020).

We used our method to study clonal structure, infer selection coefficients for all mutant alleles, and compute fitness values for eight genotypes with measured TOB MICs. Supplementary material fig. S9, Supplementary Material online, shows the reconstructed clade frequencies, allele frequency covariance matrix, and performance on inferring fitness compared with three other methods. Here, our method and *Evoracle* provide the best correlation between inferred genotype fitness and measured MICs.

## Discussion

Here we proposed a computational method to reconstruct clonal structure from time-series allele frequency data. Evaluation on simulated data shows that it accurately recovers the covariance information from time-series allele frequencies, and, when used with MPL, improves the inference of the fitness effects of individual mutations. We then applied our approach to several experimental data sets, finding clonal structure in Lenski’s LTEE and in other microbial evolution experiments. Importantly, tests on the LTEE data with large numbers of alleles show that our method requires 1–2 orders of magnitudes less run time than two alternative methods of haplotype reconstruction (supplementary material fig. S10, Supplementary Material online), allowing us to study data sets that would be impossible with other approaches. However, the haplotype reconstruction problem considered by the two alternative methods is also more computationally challenging than the clade reconstruction task that we consider. This difference should be kept in mind when considering the computational performance of each method.

While we expect that our approach should be applicable to a wide range of data sets, some features may be difficult to incorporate. We assume that populations do not undergo recombination, and violation of this assumption would make it challenging to sort sequences into nonoverlapping clades. By introducing multiple competition periods, our method can capture cases with hierarchical clonal structure, when a single clade (i.e. the clade that dominates the population at the end of a period) branches into sub-clades for each period. However, when multiple coexisting clades branch into sub-clades simultaneously, our method may not infer all the details of the sub-clonal structure.

Overall, our method aims to reveal the evolutionary dynamics at an intermediate granularity, between completely ignoring LD and fully reconstructing all haplotypes. Specifically, our method reconstructs clades (collections of alleles with correlated frequencies) and their time-series frequencies, from which pairwise LD can be computed and used for inference of selection coefficients. However, the reconstruction of specific haplotypes and their time-series frequencies are not inferred at this level. An advantage of approaching this problem at an intermediate level is that our method maintains accuracy while reducing computational costs. This allows us to apply our method to data featuring a large number of alleles and a high degree of sampling noise. Methods that reconstruct full haplotype information (Franssen et al. 2017; Barghi 2019; Deitrick 2020; Pelizzola et al. 2021; Shen et al. 2021; Li and Barton 2023), however, can potentially reveal the dynamics of evolution at a finer level.

Future work could extend the method that we have described here. First, when such data are available, longer reads that cover multiple polymorphisms could be used to place stronger constraints on clonal structure. Similarly, other sources of prior knowledge could also be incorporated into the fitting procedure to generate clades. Incorporating “soft” clade identities, where a mutation can be associated in varying degrees with more than one clade, could also extend the viability of our approach to systems with frequent recombination. The method that we use to infer the fitness effects of mutations could also be extended in different ways. Past work has considered the inference of pairwise epistasis (Sohail et al. 2022), but not global “diminishing returns” epistasis (Johnson et al. 2023). The frequent observation of diminishing returns epistasis in experiments (Rokyta et al. 2009; Wiser et al. 2013; Kryazhimskiy et al. 2014; Jerison 2017) would make this a logical baseline fitness model in future work. Other natural extensions to the model include selection that is frequency-dependent or time-varying.

## Supplementary Material

msae060_Supplementary_Data
