# Supplementary material for: Correlated Allele Frequency Changes Reveal Clonal Structure and Selection in Temporal Genetic Data
Source: Mol Biol Evol. 2024 Mar 20;41(4):msae060. doi: 10.1093/molbev/msae060 (PMC10986812; doi:10.1093/molbev/msae060)
Supplement: msae060_Supplementary_Data [file msae060_supplementary_data.pdf]

Methods

Evolutionary model

We assume a WF model consisting of N individuals evolving under mutation and selection. Each individual is represented by a sequence of length L. The loci are assumed to be bi-allelic where the value of each locus is either 0 (wild-type (WT)) or 1 (mutant), thus resulting in $M = 2^L$ possible haplotypes. For clarity, we use $i, j, ...$ to refer to locus indices and $a, b, ...$ to refer to haplotype indices. The index is shown as a subscript when representing only one of the locus or haplotype indices. However, when both indices need to be shown simultaneously, the locus index is shown as a subscript while the haplotype index is shown as a superscript. Let $n_a(t)$ denote the number of individuals in the population that belong to haplotype a at generation t such that $\sum_{a=1}^{M} n_a(t) = N$. At generation t, denote $\boldsymbol{z}(t) = (z_1(t), ..., z_M(t))$ as the observed haplotype frequencies with $z_a(t) = \frac{n_a(t)}{N}$. The observed allele frequencies are correspondingly $\boldsymbol{x}(t) = (x_1(t), ..., x_L(t))$ and are related to haplotype frequencies by $x_i = \sum_a g_i^a x_a$ where g_i^a represents the allele (either 0 or 1) at the ith locus of the ath gentype.

Here we assume that the fitness contribution from individual alleles is additive, such that the fitness f_a of the ath haplotype can be written

$$f_a = 1 + \sum_{i=1}^{L} s_i g_i^a \,. \tag{3}$$

Here the s_i denote the time-invariant selection coefficients for mutant alleles, which quantify the selective advantage of mutant allele i relative to wild-type (WT). This model is consistent with a multiplicative fitness model where the effects of individual mutations are small, or an exponential fitness function for an additive trait, where each mutation is assumed to make a small contribution to the trait value.

Under Wright-Fisher dynamics, the probability of observing haplotype frequencies $\boldsymbol{z}(t+1)$ at generation $t+1$, given haplotype frequencies of $\boldsymbol{z}(t)$ at generation t, is

$$P(\boldsymbol{z}(t+1)|\boldsymbol{z}(t)) = N! \prod_{a=1}^{M} \frac{p_a(\boldsymbol{z}(t))^{N z_a(t+1)}(\boldsymbol{n})}{(N z_a(t+1))!} \tag{4}$$

where $p_a(\boldsymbol{z}(t))$ is the probability of observing haplotype a at generation t. To derive this expression, we sum over contributions to generating haplotype a in the next generation, including the effects of mutation, recombination, and selection,

$$p_a(\boldsymbol{z}(t)) = \frac{z_a(t) f_a + \sum_{b|b \neq a} (\mu_{ba} z_b(t) f_b - \mu_{ab} z_a(t) f_a)}{\sum_{b=1}^{M} z_b(t) f_b} \,. \tag{5}$$

Here μ_{ba} is the probability for haplotype b to mutate to haplotype a in a generation.

We assume that mutations at different loci are independent with the same mutation rate μ, so that $\mu_{ba} = \mu^{d(b,a)}(1-\mu)^{L-d(b,a)}$ where $d(b,a)$ is the number of different alleles (Hamming distance) between haplotype b and haplotype a.

Below, we will follow the assumption that the population size N is large, and that the selection coefficients s_i, mutation rate μ, and recombination rate r are small ($\mathcal{O}(1/N)$). For now, expanding the expression for $p_a(\boldsymbol{z}(t))$ to eliminate terms of order μ^2 and higher, we have

$$p_a(\boldsymbol{z}(t)) = \frac{z_a(t) f_a + \mu \sum_{b|d(b,a)=1} (z_b(t) f_b - z_a(t) f_a)}{\sum_{b=1}^{M} z_b(t) f_b} \tag{6}$$

The probability that the haplotype frequency vector follows a particular evolutionary path $(\boldsymbol{z}(t_1), \boldsymbol{z}(t_2), \ldots, \boldsymbol{z}(t_K))$, conditioned on the initial state $\boldsymbol{z}(t_0)$, population size N, mutations rate μ and selection coefficients $\boldsymbol{s}$ is

$$P\left((\boldsymbol{z}(t_k))_{k=1}^{K} | \boldsymbol{z}(t_0), N, \mu, \boldsymbol{s} \right) = \prod_{k=0}^{K-1} P(\boldsymbol{z}(t+1)|\boldsymbol{z}(t)) \tag{7}$$

Weighting observed products of allele frequency changes

For finitely sampled data, variations in allele frequencies near the extremes (0 and 1) are more susceptible to sampling noise. To reduce the influence of noise, we weight the entries of $\boldsymbol{\Delta x \Delta x}$ according to the smaller of the two allele frequency variances at each time. Specifically, the weight matrix is

$$W_{ij}(t_k) = \min(v_i(t_k), v_j(t_k)) \,,$$

where $v_i(t_k)$ is the variance of interpolated frequency of allele i in the middle of time point t_k and t_{k+1},

$$v_i(t_k) = \frac{x_i(t_k) + x_i(t_{k+1})}{2} \left(1 - \frac{x_i(t_k) + x_i(t_{k+1})}{2} \right) .$$

We denote the sum of the weighted $\boldsymbol{\Delta x \Delta x}$ matrix over an entire evolutionary history as D,which has entries

$$D_{ij} = \sum_{k=1}^{K-1} \frac{1}{\Delta t_k} \boldsymbol{\Delta x \Delta x}_{ij}(t_k) W_{ij}(t_k)$$

where Δt_k is the number of generations between sampling time points t_k and t_{k+1}, i.e., $\Delta t_k = t_{k+1} - t_k$. The factor of $1/\Delta t_k$ arises from linearly interpolating allele frequency trajectories, and summing $\boldsymbol{\Delta x \Delta x}$ entries from generation to generation. To prevent confusion, here we note that the D matrix defined above is different from the LD matrix[38], which is also commonly written as D.

Forming initial clades

To determine which clade an allele i should belong to, we first determine the group that has the largest cooperation score with it, which we denote $g_i^{\max}$,

$$g_i^{\max} = \underset{g}{\operatorname{argmax}}\, \rho_{\text{coop}}(i, g) \,.$$

We quantify the degree to which allele i should be assigned to group $g_i^{\max}$ by a confidence score, $\rho_{\mathrm{conf}}(i)$, computed as its cooperation score with group $g_i^{\max}$ minus the sum of its cooperation scores with all other groups,

$$\rho_{\mathrm{conf}}(i) = \rho_{\mathrm{coop}}(i, g_i^{\max}) - \sum_{g \neq g_i^{\max}} \rho_{\mathrm{coop}}(i, g) \,.$$

With the above concepts, we form the initial clades by first identifying the pair of alleles with most competing behavior, i.e., the ones with the most negative entry in D, and assign them as two initial groups. We then select the next allele i out of the remaining ones that has the highest confidence score, $\rho_{\mathrm{conf}}(i)$. The selected allele i is processed in one of the following ways.

1. We assign it to group $g_i^{\max}$ if it cooperates with group $g_i^{\max}$ and competes with all other groups. That is, $\rho_{\mathrm{coop}}(i, g_i^{\max}) > 0$, and $\rho_{\mathrm{coop}}(i, g) \leq 0$ for all other groups.

2. We mark it as a shared allele if it cooperates with more than one group, such that there are multiple groups with $\rho_{\mathrm{coop}}(i, g) > 0$.

3. We assign it to a new group if it competes with all existing groups, with $\rho_{\mathrm{coop}}(i, g) \leq 0$.

We repeat this procedure of selecting and processing alleles iteratively until all alleles have been processed.

Denoting the number of groups as n, we put the group of shared alleles as the first group (group 1), and sort the other groups (groups 2 to n) by their sizes from largest to smallest. Considering possible redundant groups in the end with negligible signals, we select groups 2 to m so that their entries in the D matrix are just enough to account for more than a percentage (set to 99%) of all non-shared alleles in terms of the sum of absolute values, i.e., we select the smallest m such that

$$\frac{\sum_{i \neq j \in \{\mathrm{group}\,2,\ldots,m\}} |D_{ij}|}{\sum_{i \neq j \in \{\mathrm{group}\,2,\ldots,n\}} |D_{ij}|} \geq 99\% \,.$$

Each of groups 2 to m then constitutes an initial clade.

Iterative refinement of clade membership and frequencies

Below, we first introduce the concept of a "competition period," a time during which a specific set of clones compete, which is needed to account for cases with time-varying clonal structures. For each competition period, we obtain an initial estimate of clade frequencies from their constitutive allele frequencies. We then iteratively refine clonal identities and clade frequencies until convergence: We detect improbable classifications of alleles and re-classify them to appropriate clades, with clade frequencies re-estimated correspondingly, until no more improbable cases are detected. Finally, we merge our estimates for clade identities and frequencies from all competition periods.

Dividing evolution into competition periods

During the course of evolution, a population can exhibit different patterns of clonal interference at different times. For example, consider a population that consists of multiple, competing clades until one of the clades outcompetes the others and fixes. At a later time, new beneficial mutations can arise and compete with one another, initiating another period of clonal interference. To account for these possibilities, we define a "competition period" as a period of time bounded by the beginning and end of the coexistence of several clades. When an evolutionary history consists of multiple competition periods, each period can have a distinct clonal structure, i.e., number of clades, clade frequency trajectories, and assignment of alleles to different clades. It is thus important to detect and separate these competition periods to infer this information correctly.

We detect the existence of multiple competition periods and set approximate boundaries for them by identifying fixation events of mutations involved in clonal interference. For each clade (excluding the shared alleles) generated in the clustering step, we identify its member alleles that fix during the evolution. An allele i is identified as fixed at time point t_k if its mean frequency after t_k exceeds a threshold value, set here to 0.98. We take the fixation time of the allele with the largest confidence score, $\rho_{\mathrm{conf}}(i)$, as the fixation time of that clade. The fixation of a clade then marks the boundary between two successive competition periods. When there are n groups that fix during the evolution, we divide the whole evolutionary history into $n+1$ competition periods at their fixation times, and infer the clonal structure for each competition period separately.

The initial clades determined in the previous section are formed by analyzing the matrix D integrated over the entire evolution. During a competition period, some of these clades may have become extinct or may be yet to emerge. The population can therefore exhibit different patterns of clonal interference in different competition periods. To infer the clonal structure for each competition period, we form initial clades again for each competition period separately. The steps to form initial clades for a competition period are the same as introduced in the previous section, except that the matrix D is integrated over that period alone. By limiting the integration of matrix D to each period, mutations that fixed before a period or have yet to appear will not interfere with the classification of mutations that are at intermediate frequencies within that period. This reduces noise in the integrated D matrix and could make clustering more accurate.

Estimating clade frequencies

To estimate the frequencies of these clades, we select alleles that can provide reliable information on clade frequencies. We filter out alleles that are shared by multiple groups in the clustering process, alleles that fix during the competition period, and alleles that get extinct during the evolution. An allele i is identified as extinct at time point t_k if its mean frequency after t_k is lower than a threshold value (set to 1%). These alleles are excluded from the next step

because they provide less reliable information to distinguish between clades. The remaining alleles are then used to obtain an initial estimate of clade frequencies.

At time point t_k, an allele in clade c can be in one of three states: fixed within its clade, at an intermediate frequency, or yet to emerge. With sampling noise, frequencies of alleles that are fixed within clade c are distributed around the true underlying clade frequency trajectory, $f_c(t_k)$. Frequencies of alleles that have not emerged are near zero. We assume that the typical time required for alleles to fix within a clade is shorter than the time for entire clades to fix in the population. Therefore, at most time points, most alleles have either already fixed within clades or have not yet emerged. Equivalently, if we view frequencies of all alleles in clade c as sampled from a distribution, then its probability density should have two peaks at zero and $f_c(t_k)$ at most time points. Based on this assumption, we estimate the frequency of clade c at time point t_k by frequencies of all its member alleles that exceed a threshold (0.01), $S = \{x_i(t_k) \mid \text{allele } i \in \text{clade } c,\ x_i(t_k) > 0.01\}$, in three steps.

1. We consider only frequencies larger than a minimum threshold (0.01). When there are no such frequencies, the clade frequency is set as the mean of all its member frequencies.

2. We estimate the probability density of frequencies in set S using a kernel density estimation (KDE) method[39]. When set S contains too few frequencies to yield a reliable estimate (fewer than four frequencies), we resort to setting the clade frequency as the mean of all frequencies in set S.

3. We identify the frequency with the highest estimated probability density, select frequencies that fall within a window of width 0.2 around that frequency, and take their mean frequencies as an initial estimate of the clade frequency $f_c(t_k)$.

Refining clonal identities and clade frequencies

The initial estimates of clonal identities and clade frequencies are based on the clustering results, which are based, in turn, on the evaluation of co-varying behaviors quantified by ρ_{coop}. Here we refine these estimates by accounting for sampling probabilities. Without sampling noise, the frequency of an allele should not exceed its clade frequency f_c. However, this becomes possible with noise from finite sampling. In cases where an allele i has a higher frequency than its clade at a time point t_k, $x_i(t_k) > f_c(t_k)$, we assume that its true frequency is the clade frequency $f_c(t_k)$, and quantify the sampling probability by a binomial distribution, i.e.,

$$P(x_i(t_k)|R_i(t_k), f_c(t_k)) = \binom{R_i(t_k)x_i(t_k)}{R_i(t_k)} f_c(t_k)^{R_i(t_k)x_i(t_k)}(1-f_c(t_k))^{R_i(t_k)(1-x_i(t_k))}$$

where $R_i(t_k)$ is the read depth of the data at allele i at time point t_k, and $f_c(t_k)$ is the estimated frequency of clade c at time point t_k. Note that these probabilities are only considered for alleles that have a higher frequency than their clades at some time point.

We refine estimates of clonal identities and clade frequencies in a recursive way. In each round of refinement, for every allele with a frequency higher than its current clade, we compute the logarithm of its mean sampling probabilities with respect to all clades over the competition period, i.e.,

$$\log \bar{P}(i,c) = \frac{1}{T}\sum_{t_k} \log P\left(x_i(t_k)|R_i(t_k), f_c(t_k)\right) \qquad \textbf{(8)}$$

where T is the number of time points in the current competition period. The clade it most probably belong to based on sampling probabilities is then denoted as

$$c_i^{\max} = \underset{c}{\operatorname{argmax}} \log \bar{P}(i,c)\,.$$

We then reclassify it to the clade $c_i^{\max}$ when the probability gain by doing this is larger than a threshold η, i.e,

$$\log \bar{P}(i, c_i^{\max}) - \log \bar{P}(i, c_i^{\text{current}}) > \eta\,. \qquad \textbf{(9)}$$

After each round of refinement, we re-estimate time-series frequencies for all clades according to refined clonal identities following the steps outlined in the previous section. We then start another round of refinement, unless there is no reclassification performed in the last round.

Incorporating initially excluded alleles

During the previous steps of estimating clade frequencies and refining clades and frequency estimates, we exclude alleles that provide less reliable information to distinguish between clades. These are alleles that are shared by multiple groups in the clustering process, alleles that fix during the competition period, and alleles that never reach a minimum frequency threshold throughout the competition period. After we have refined clades and their frequency estimates, we reconsider these excluded alleles by evaluating their cooperation scores and sampling probabilities with all clades. The cooperation score between an allele i and clade c is defined as

$$\rho(i,c) = \sum_k \frac{1}{\Delta t_k} \Delta x_i(t_k) \Delta f_c(t_k) w(i,c)\,, \qquad \textbf{(10)}$$

where $\Delta f_c(t_k) = f_c(t_{k+1}) - f_c(t_k)$, and $w(i,c) = \mathrm{min(x_i(t_k)(1-x_i(t_k)), f_c(t_k)(1-f_c(t_k)))}$.

Similar to entries in the matrix D, which quantify correlated frequency changes for different alleles, the cooperation score $\rho(i,c)$ quantifies correlated frequency changes of allele i and clade c. We denote c_i^{maxCoop} as the clade with highest cooperation score with allele i, and c_i^{maxProb} as the clade with highest mean sampling probability with allele i, i.e., $c_i^{\text{maxProb}} = \underset{c}{\operatorname{argmax}} \log \bar{P}(i,c)$. We assign allele i to clade c_i^{maxCoop} when the two following conditions are met.

1. $\rho(i, c_i^{\text{maxCoop}}) > \tilde{\rho}(c_i^{\text{maxCoop}})$, where $\tilde{\rho}(c_i^{\text{maxCoop}})$ represents the median cooperation score with clade c_i^{maxCoop} for all its member alleles.

2. $\log \bar{P}(i, c_i^{\mathrm{maxProb}}) - \log \bar{P}(i, c_i^{\mathrm{maxCoop}}) <= \eta$, where η is a preset threshold, and $\log \bar{P}$ terms are averaging logarithm of sampling probabilities over time points at which frequency of allele i exceeds frequency of clade c.

Otherwise, we keep allele i as a non-clonal allele which is shared across clades. The first condition ensures that allele i exhibits co-varying behaviors to a greater extent than at least half of member alleles of clade c_i^{maxCoop}. The second condition prevents an assignment that is too improbable based on sampling probabilities.

Merging results from all competition periods

After recovering the clonal structure for each competition period, we merge results from all periods by aggregating clonal identities and connecting clade frequencies. For two successive competition periods p and $p+1$, one of the clades in period p dominates the population at some time, becomes the "ancestor" clade in period $p+1$, and develops into sub-clades. We denote this clade as c_a, and its fixation time as t_f. When the first sub-clade of c_a emerges after t_f, the clonal dynamics of periods p and $p+1$ are well separated in time, and their clonal structures can be directly merged, e.g., as shown in **Supplementary Fig. S1A**. However, when the first sub-clade of c_a emerges before t_f, say at $t_e < t_f$, there is an overlap period $[t_e, t_f]$ when c_a has not fixed and a sub-clade has started to emerge. In such a scenario, the frequency trajectory of this sub-clade during this overlap is not estimated in period $p+1$, since we divide these two competition periods at the fixation time t_f. It may not be identified in period p, as the signal of it in the D matrix integrated in that period can be weak. In order to estimate this segment of clade frequency trajectory, we make use of an alternative way to define competition periods. Instead of dividing at fixation times of clades, we set boundaries for competition periods as time points when the clade that is going to fix (i.e., the ancestor clade of the next competition period) first breeds a sub-clade. Such boundaries can be determined because we have already inferred clonal structure for these competition periods. With re-defined boundaries, we recover again the clonal structure for each competition period. With results from both rounds, we can now connect clade frequencies seamlessly across competition periods. A schematic example of this workflow is shown in **Supplementary Fig. S1B**.

Marginal path likelihood (MPL) inference

Sohail et al. presented a diffusion approximation and path integral expression for the stochastic haplotype frequency dynamics under the WF model setting[40]. The path integral expression for the probability of observing a trajectory of haplotype frequencies $(\boldsymbol{z}(t_1), \boldsymbol{z}(t_2), \ldots, \boldsymbol{z}(t_K))$ is given by

$$\begin{aligned} P&\left((\boldsymbol{z}(t_k))_{k=1}^{K} | \boldsymbol{z}(t_0), N, \mu, \boldsymbol{s}\right) \\ &= \prod_{k=0}^{K-1} P\left(\boldsymbol{z}(t+1) | \boldsymbol{z}(t)\right) \\ &\approx \left(\prod_{k=0}^{K-1}\left[\frac{1}{\sqrt{\det C(\boldsymbol{z}(t_k))}}\left(\frac{N}{2\pi\Delta t_k}\right)^{M/2} d\boldsymbol{z}(t_{k+1})\right]\right) \\ &\quad \times \exp\left(-\frac{N}{2} S\left((\boldsymbol{z}(t_k))_{k=0}^{K}\right)\right) \end{aligned} \quad \textbf{(11)}$$

where $C(\boldsymbol{z}(t_k))$ is the haplotype covariance matrix, i.e.,

$$(C(\boldsymbol{z}(t)))_{ab} := \begin{cases} z_a(t)(1-z_a(t)) & a = b \\ -z_a(t) z_b(t) & a \neq b \end{cases}. \quad \textbf{(12)}$$

and

$$\begin{aligned} S&\left((\boldsymbol{z}(t_k))_{k=0}^{K}\right) \\ &= \sum_{k=0}^{K-1} \frac{1}{\Delta t_k} \sum_{a=1}^{M}\sum_{b=1}^{M} \left[z_a(t_{k+1}) - z_a(t_k) - \Delta t_k d_a(\boldsymbol{z}(t_k))\right] \\ &\quad \times \left(C^{-1}(z(t_k))\right)_{ab} \left[z_b(t_{k+1}) - z_b(t_k) - \Delta t_k d_b(\boldsymbol{z}(t_k))\right] \end{aligned}$$

where $d_a(\boldsymbol{z}(t_k))$ is the expected change in haplotype frequency z_a at time t_k.

The MAP estimate of the selection coefficients can be obtained by solving

$$\hat{\boldsymbol{s}} = \underset{s}{\operatorname{argmax}}\, \mathcal{L}\left(\boldsymbol{s} | N, \mu, (\boldsymbol{z}(t_k))_{k=1}^{K}\right) P^{prior}(\boldsymbol{s}) \quad \textbf{(13)}$$

where $P^{\mathrm{prior}}(\boldsymbol{s})$ is an assumed prior

$$P^{\mathrm{prior}}(\boldsymbol{s}) = \frac{1}{(2\pi\sigma^2)^{R/2}} \exp\left(-\frac{1}{2\sigma^2}\boldsymbol{s}^T \boldsymbol{s}\right) \quad \textbf{(14)}$$

with mean zero and variance $\sigma^2 > 0$, and the likelihood function is given as

$$\mathcal{L}\left(\boldsymbol{s} | N, \mu, (\boldsymbol{z}(t_k))_{k=1}^{K}\right) = P\left((\boldsymbol{z}(t_k))_{k=1}^{K} | \boldsymbol{z}(t_0), N, \mu, \boldsymbol{s}\right) \quad \textbf{(15)}$$

The right-hand side is approximated by Equation Eq. (11). Differentiating the expression with respect to $\boldsymbol{s}$ and equating to zero leads to the MAP estimator of selection coefficients s

$$\begin{aligned} \hat{\boldsymbol{s}} &= \left[\sum_{k=0}^{K-1} \Delta t_k C\left(\boldsymbol{x}(t_k)\right) + \gamma I\right]^{-1} \\ &\quad \times \left[\boldsymbol{x}(t_k) - \boldsymbol{x}(t_0) + \mu \sum_{k=0}^{K-1} \Delta t_k \left(2\boldsymbol{x}(t_k) - 1\right)\right] \end{aligned}$$

where $\gamma = 1/N\sigma^2$.

Simulation

To benchmark the performance of our method, we generate artificial time series sequence data by simulating evolution as a Wright-Fisher process. As in the previous section, the loci are assumed to be bi-allelic. In the default setup, in each of the 40 simulations that we ran, a population of $N = 1000$ sequences started with all wild-type sequences and evolved for $T = 1000$ generations. At a generation t, the population first goes through a multinomial sampling process to determine the number of sequences $z_a(t)$ for each existing haplotype a in the current generation, where the probability p_a for a haplotype a to be drawn is proportional to the product of its frequency in the last generation $z_a(t-1)$ and fitness f_a, i.e.,

$$p_a(\boldsymbol{z}(t)) = \frac{z_a(t-1)f_a}{\sum_{b=1}^{M} z_b(t-1)f_b} \qquad \textbf{(16)}$$

Each sequence, at each generation, has a fixed probability (2×10^{-4}) to acquire a new mutation. We assume that at most one mutation occurs in a given sequence. We enforce an additional condition that promotes the occurrence and persistence of clonal interference: Each mutation occurs on a unique site, which also means that each mutation can only be acquired once. The selection coefficient of each mutation is drawn from a Gaussian distribution centered at 0.03 with a standard deviation of 0.01. At the end of the simulation, only alleles whose frequencies once exceed a threshold of 0.05 are preserved and considered in later analysis.

We varied the default simulation setup to test our method more thoroughly. We considered simulations with smaller ($N = 100$) or larger ($N = 10000$) population sizes, simulations with rare recombination events, simulations starting from a mixture of random haplotypes, and simulations of diploid populations with random mating. In the diploid population, characterized by having two sets of chromosomes, we correspondingly adjusted the mutation rate to be twice as large (4×10^{-4}).

Supplementary results

Comparing runtime of methods on LTEE data

We applied the *Lolipop* and *Evoracle* methods on the LTEE data and compared their run times with our method in **Supplementary Fig. S10**. The LTEE data consists of 12 populations. Six populations are nonmutator populations with fewer than 1,000 alleles, while the other six are mutator populations with more than 1,000 alleles. When applied on the mutator populations, *Lolipop* and *Evoracle* did not finish running after 2 weeks (after which time running jobs were killed), while our method requires less than 1 day to complete. **Supplementary Fig. S10** shows that the run time of our method is typically around an order of magnitude faster than *Lolipop* and around two orders of magnitude faster than *Evoracle* for these large data sets.

Clustering results for population p3 of LTEE data

Population p3 involves more than 6,000 mutant alleles and is one of the six mutator populations in the LTEE data. We found that the dynamics of this population is better explained when we split the whole evolution into two competition periods at generation 35,000, infer for each period, and combine the inferred dynamics of two periods. We plot the conjugate clustering results in **Supplementary Fig. S8A**. Inference on each period identifies strong competition between two clades, where the first period features competition between clade 1 and clade 2, and the second period features competition between clade 3 and clade 4. When looking at their relationships, we found that there are 21 mutant alleles that are shared between either clade in period 1 and either clade in period 2. Their frequency trajectories (plotted in yellow in **Supplementary Fig. S8A**) follow either clade 1 or clade 2 in the first period, and then follow either clade 3 or clade 4 in the second period. This suggests complex evolutionary dynamics in which the same mutations appear or arise on different backgrounds.

Data and code

Data and code used in our analysis is available in the GitHub repository at https://github.com/bartonlab/paper-clonal-dynamics. This repository also contains Jupyter notebooks that can be run to reproduce these results.

References

38. Hedrick, P. W. Gametic disequilibrium measures: proceed with caution. *Genetics* **117**, 331–341 (1987).
39. Scott, D. W. *Multivariate Density Estimation: Theory, Practice, and Visualization* (John Wiley & Sons, 2015).
40. Sohail, M. S., Louie, R. H. Y., McKay, M. R. & Barton, J. P. MPL resolves genetic linkage in fitness inference from complex evolutionary histories. *Nat. Biotechnol.* **39**, 472–479 (2021).
41. Good, B. H., McDonald, M. J., Barrick, J. E., Lenski, R. E. & Desai, M. M. The dynamics of molecular evolution over 60,000 generations. *Nature* **551**, 45–50 (2017).

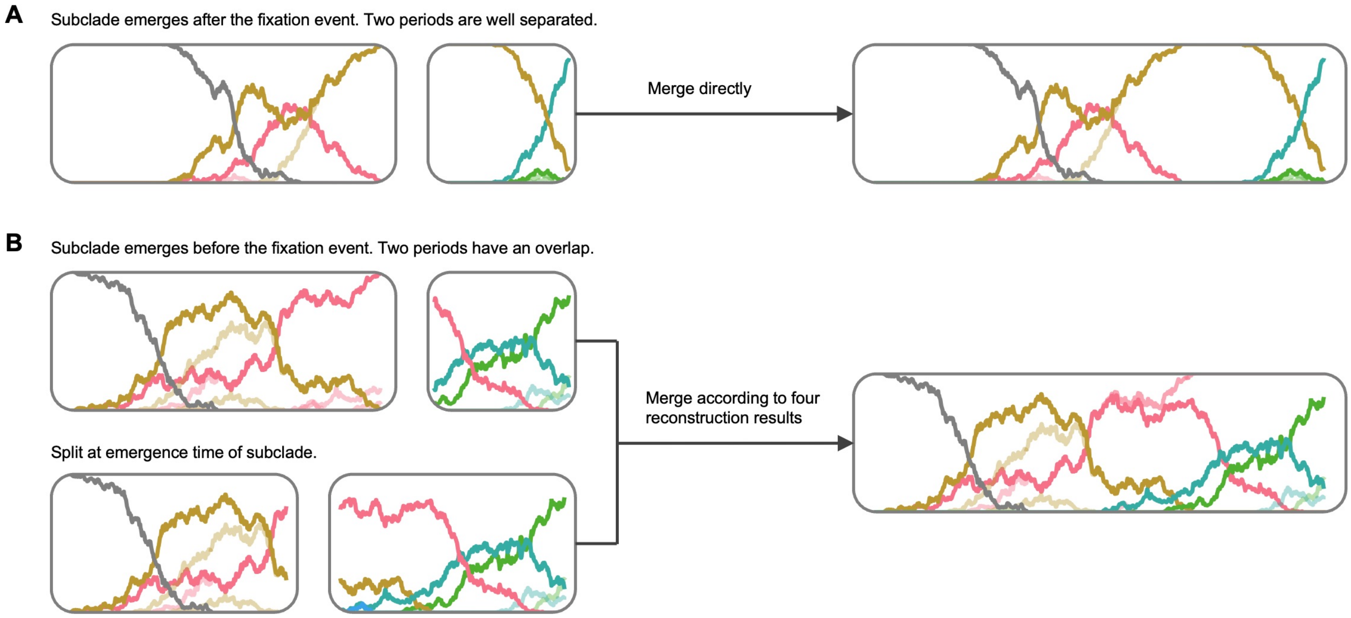

Supplementary Fig. S1. Merging results from two successive competition periods. **A**, When subclades in the latter period emerge after the dominant clade in the former period gets fixed, two periods are well separated in time, and can be merged directly. **B**, When subclades in the latter period emerge before the dominant clade in the former period gets fixed, two periods have an overlap. The frequency trajectories of emerging subclades are not complete (upper left plot in **B**). In order to estimate frequency trajectories of emerging subclades in the latter period, we perform another alternative split, where we split the whole period at the emergence time of the first emerging subclade. Upon identifying clade members and estimating clade frequencies for the four periods, we combine results from all four periods to form a complete reconstruction. The allele frequency trajectories plotted in the figure are from one case of simulated data, where a population of 1,000 sequences were simulated to evolve for 1,000 generations.

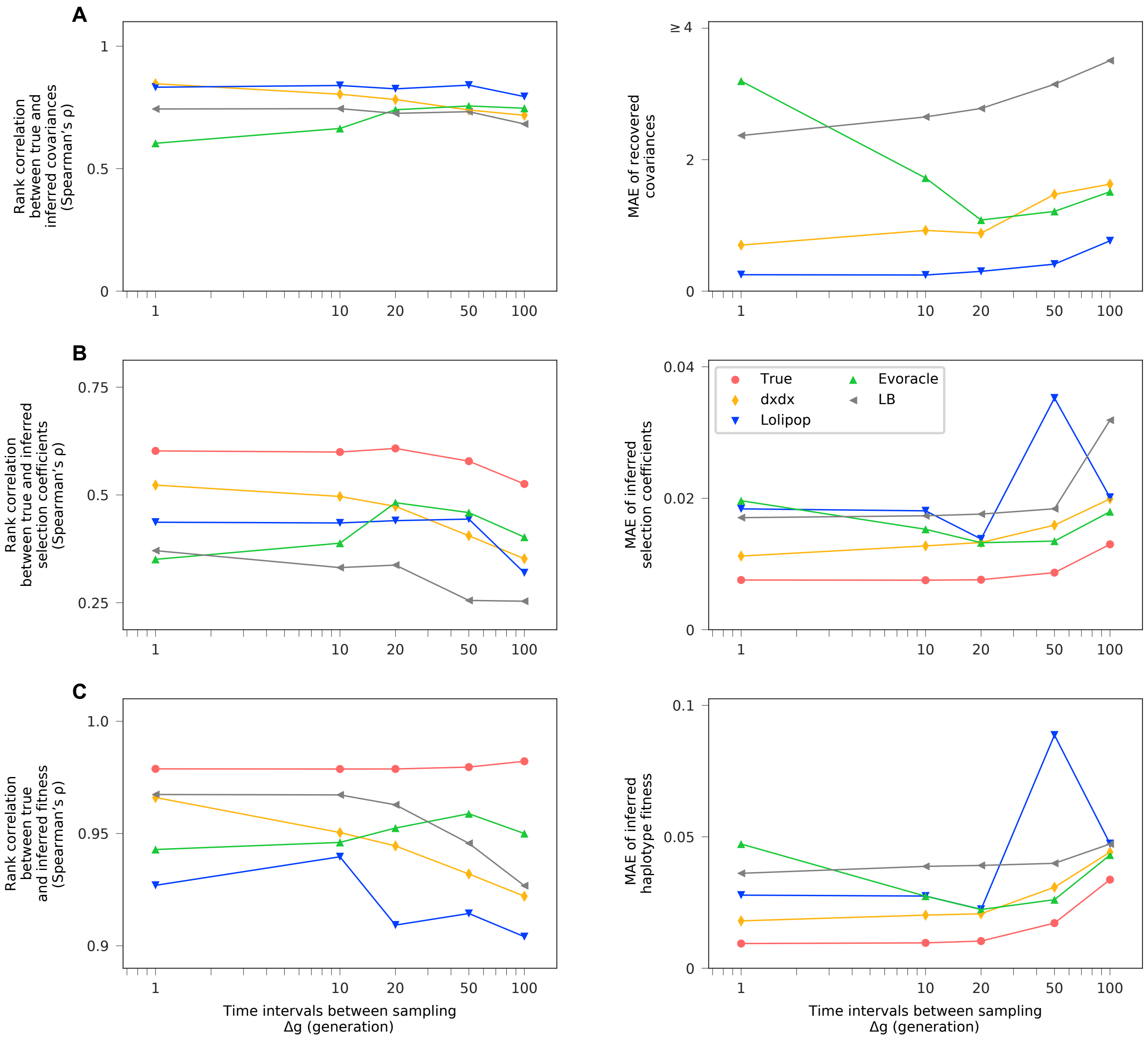

Supplementary Fig. S2. Effects of different sampling temporal intervals. Performance of five methods averaged over 40 simulations with different sampling temporal intervals for inference of (**A**) integrated allele frequency covariance, (**B**) selection coefficients, and (**C**) haplotype fitness. The left column shows rank correlations with true values, and the right column shows the mean absolute error (MAE) of inferred values versus true values. The *True* method uses the true allele frequency covariance matrix, which is not available in short read data, and represents the ideal performance.

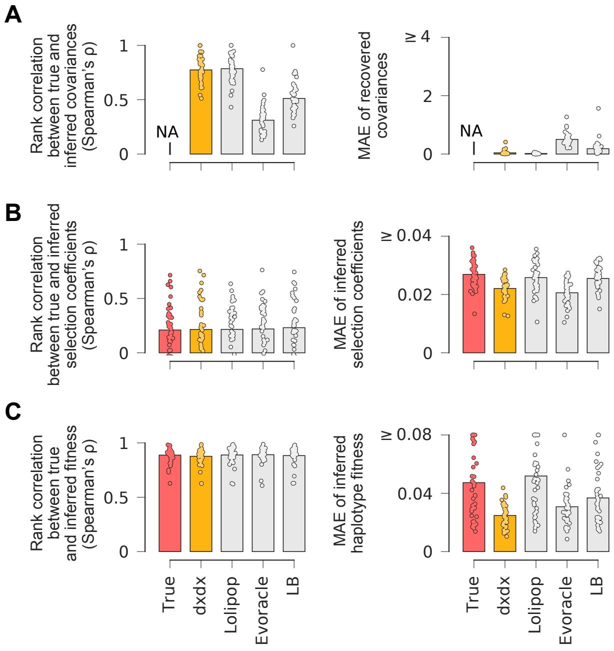

Supplementary Fig. S3. Benchmarking on simulated data with a population size of 100. Performance of five methods for inference of (**A**) integrated allele frequency covariance, (**B**) selection coefficients, and (**C**) haplotype fitness. The left column shows rank correlations with true values, and the right column shows the mean absolute error (MAE) of inferred values versus true values. The *True* method uses the true allele frequency covariance matrix, which is not available in short read data, and represents the ideal performance.

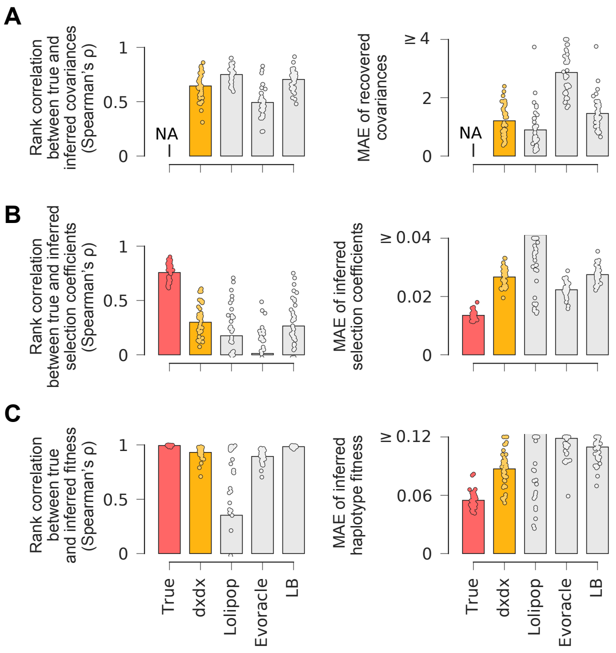

Supplementary Fig. S4. Benchmarking on simulated data with a population size of 10000. Performance of five methods for inference of (**A**) integrated allele frequency covariance, (**B**) selection coefficients, and (**C**) haplotype fitness. The left column shows rank correlations with true values, and the right column shows the mean absolute error (MAE) of inferred values versus true values. The *True* method uses the true allele frequency covariance matrix, which is not available in short read data, and represents the ideal performance.

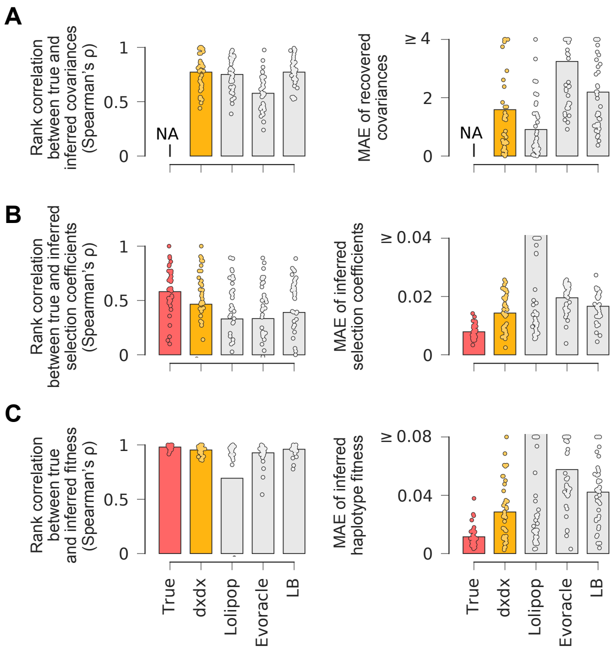

Supplementary Fig. S5. Benchmarking on simulated data with rare recombination. Performance of five methods for inference of (**A**) integrated allele frequency covariance, (**B**) selection coefficients, and (**C**) haplotype fitness. The left column shows rank correlations with true values, and the right column shows the mean absolute error (MAE) of inferred values versus true values. The *True* method uses the true allele frequency covariance matrix, which is not available in short read data, and represents the ideal performance.

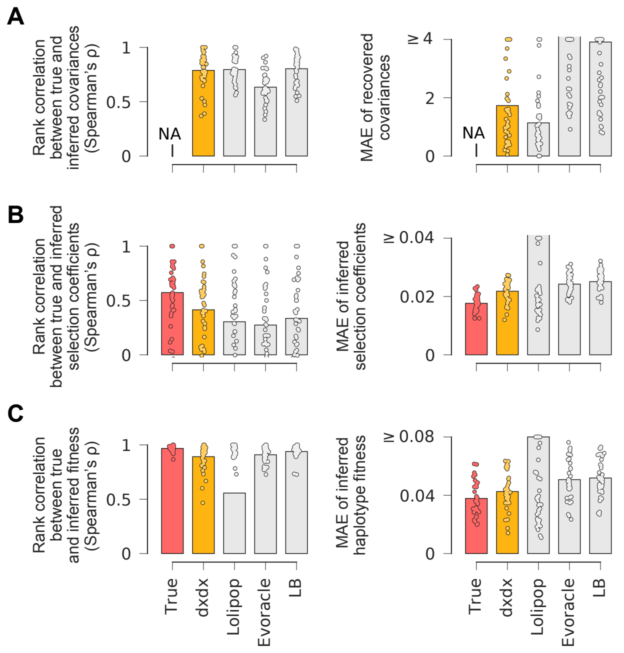

Supplementary Fig. S6. Benchmarking on simulated data for a diploid population with random mating. Performance of five methods for inference of (**A**) integrated allele frequency covariance, (**B**) selection coefficients, and (**C**) haplotype fitness. The left column shows rank correlations with true values, and the right column shows the mean absolute error (MAE) of inferred values versus true values. The *True* method uses the true allele frequency covariance matrix, which is not available in short read data, and represents the ideal performance.

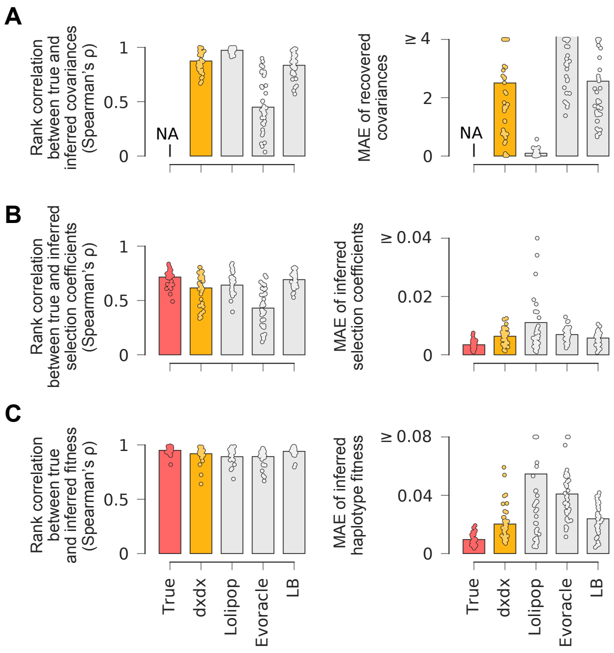

Supplementary Fig. S7. Benchmarking on simulated data starting from a random mixture of haplotypes. Performance of five methods for inference of (**A**) integrated allele frequency covariance, (**B**) selection coefficients, and (**C**) haplotype fitness. The left column shows rank correlations with true values, and the right column shows the mean absolute error (MAE) of inferred values versus true values. The *True* method uses the true allele frequency covariance matrix, which is not available in short read data, and represents the ideal performance.

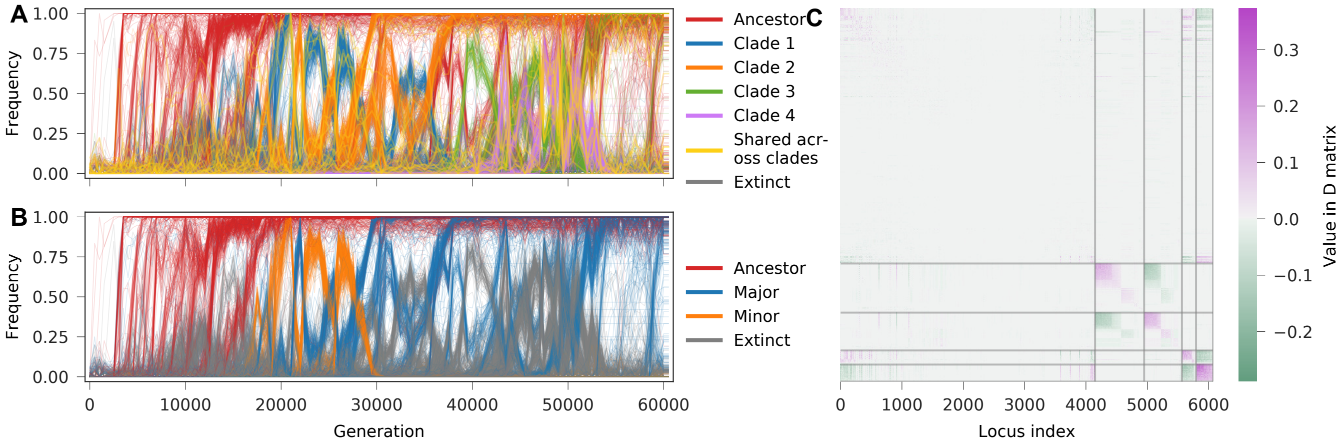

Supplementary Fig. S8. Clonal structure for population p3 of LTEE data inferred when split into two periods at generation 35,000 exhibits four-clade competition and complex dynamics. The clustering results from (**A**) our method (inferred as two periods) and (**B**) previous results[41] on the population p3 identify different patterns of clonal interference. Clades 1 and 2 are identified by inference on the first period from generation 0 to 35,000. Clades 3 and 4 are identified by inference on the second period from generation 35,000 to the last generation. For better visibility, we place trajectories of mutant alleles that are shared across clades (marked as yellow; totaling 21 alleles) at the front layer. **C**, D matrix segmented into groups according to clustering processes of the two periods.

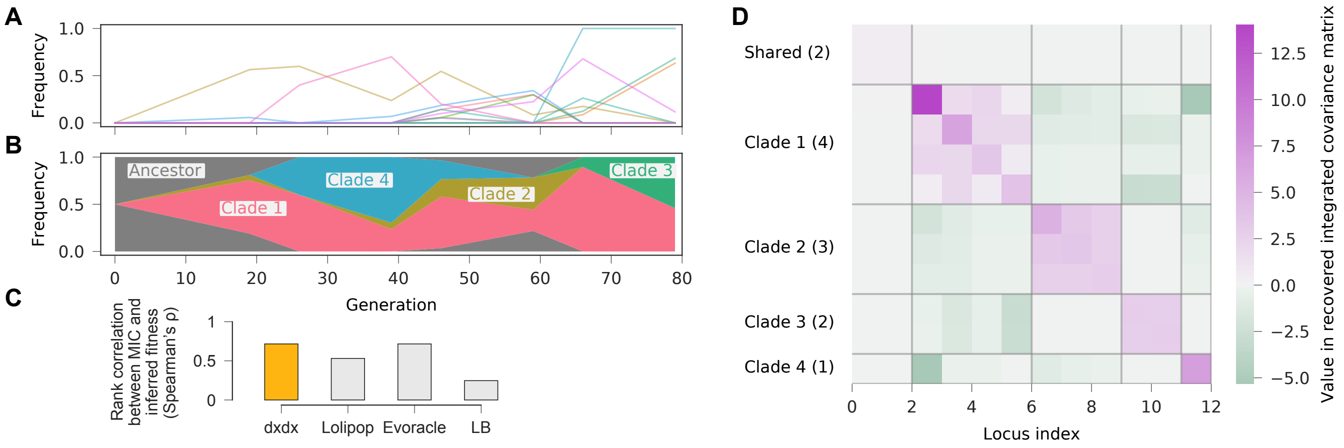

Supplementary Fig. S9. Clonal structure and fitness inference for Planktonic-2 replicate of tobramycin data. (**A**) Mutant allele frequency trajectories, and (**B**) clade frequency trajectories inferred by our method are plotted. **C**, Our method outperforms the Lolipop and LB methods, and has the same performance as Evoracle, in terms of rank correlation between inferred fitness and measured MIC values. **D**, Recovered integrated covariance matrix segmented into blocks according to clustering results of our method. The number of alleles in each clade is shown in brackets. Alleles in the same clades tend to show cooperating behaviors, as indicated by positive entries. Clades tend to show competing behaviors with each other, as indicated by negative entries.

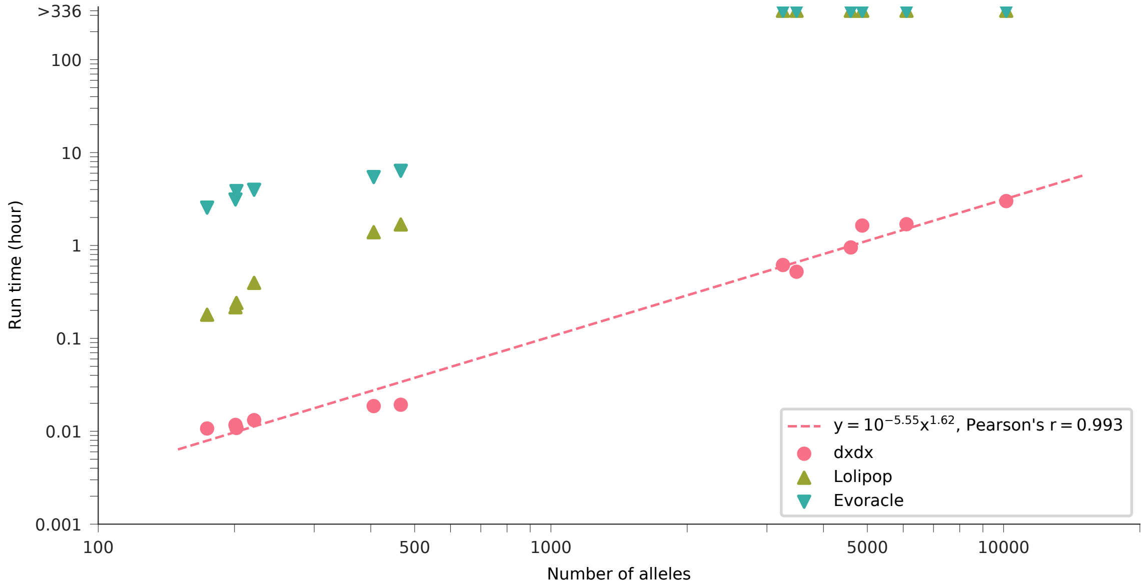

Supplementary Fig. S10. Our method requires significantly less run time on data with a large number of alleles. For mutator populations with more than 1,000 alleles, Lolipop and *Evoracle* require run times of more than 2 weeks (336 hours), while our method takes less than 1 day to complete. Overall, the run time of our method is 1 to 2 orders of magnitude less than that of *Lolipop* or *Evoracle*. A linear regression on the 12 data points for our method shows that the run time is approximately proportional to $x^{1.62}$, where x denotes the number of alleles.
